# Development and validation of an *Onchocerca ochengi* adult male worm gerbil model for macrofilaricidal drug screening

**DOI:** 10.1371/journal.pntd.0007556

**Published:** 2019-07-01

**Authors:** Fidelis Cho-Ngwa, Glory Enjong Mbah, Rene Bilingwe Ayiseh, Emmanuel Menang Ndi, Elvis Monya, Irene Memeh Tumanjong, Evans Ngandung Mainsah, Judy Sakanari, Sara Lustigman

**Affiliations:** 1 ANDI Centre of Excellence for Onchocerciasis Drug Research, Biotechnology Unit, Faculty of Science, University of Buea, Buea, Cameroon; 2 Department of Pharmaceutical Chemistry, University of California, San Francisco, California, United States of America; 3 Lindsley F. Kimball Research Institute, New York City, New York, United States of America; University of Zurich, SWITZERLAND

## Abstract

**Background:**

Onchocerciasis currently afflicts an estimated 15 million people and is the second leading infectious cause of blindness world-wide. The development of a macrofilaricide to cure the disease has been hindered by the lack of appropriate small laboratory animal models. This study therefore, was aimed at developing and validating the Mongolian gerbil, as an *Onchocerca ochengi* (the closest in phylogeny to *O*. *volvulus*) adult male worm model.

**Methodology/Principal findings:**

Mongolian gerbils (*Meriones unguiculatus*) were each implanted with 20 *O*. *ochengi* male worms (collected from infected cattle), in the peritoneum. Following drug or placebo treatments, the implanted worms were recovered from the animals and analyzed for burden, motility and viability. Worm recovery in control gerbils was on average 35%, with 89% of the worms being 100% motile. Treatment of the gerbils implanted with male worms with flubendazole (FBZ) resulted in a significant reduction (p = 0.0021) in worm burden (6.0% versus 27.8% in the control animals); all recovered worms from the treated group had 0% worm motility versus 91.1% motility in control animals. FBZ treatment had similar results even after four different experiments. Using this model, we tested a related drug, oxfendazole (OFZ), and found it to also significantly (p = 0.0097) affect worm motility (22.7% versus 95.0% in the control group).

**Conclusions/Significance:**

We have developed and validated a novel gerbil *O*. *ochengi* adult male worm model for testing new macrofilaricidal drugs *in vivo*. It was also used to determine the efficacy of oxfendazole *in vivo*.

## Author summary

Onchocerciasis is a neglected tropical disease caused by the filarial nematode *Onchocerca volvulus*. The only drug currently used for the treatment of this disease and reduction of transmission is ivermectin. However, this drug kills only the microfilarial stage of the parasite, and not the adult worm, and cases of parasite resistance to the drug have been recorded. Therefore, there is an urgent need to find an efficacious macrofilaricidal drug that targets the adult worms. To identify an effective macrofilaricide, suitable small laboratory animal models are needed for routine drug screening and/or validation *in vivo*. To develop such a model, we implanted Mongolian gerbils with adult *O*. *ochengi* male worms; *O*. *ochengi* is the closest filarial relative of *O*. *volvulus*. Gerbils appeared to be permissive to *O*. *ochengi* male worms and when treated with flubendazole, a known macrofilaricidal drug, the drug significantly decreased worm burden, motility and viability when compared to vehicle-treated animals. This model was also used to test the efficacy of oxfendazole, a drug belonging to same class as flubendazole, and it also showed potent activity on *O*. *ochengi* male worms. The gerbil *O*. *ochengi* male worm model is now available to screen new candidate macrofilaricides.

## Introduction

Onchocerciasis currently afflicts an estimated 15 million people worldwide, predominantly in Sub-Saharan Africa [1]. It is the second leading infectious cause of blindness globally [2]. Its causative agent, *Onchocerca volvulus* is transmitted by multiple bites from the infected black fly (of genus *Simulium*). The adult worms of this tissue dwelling filaria, establish in subcutaneous nodules (onchocercoma), producing millions of microfilariae (mf) which parasitize skin and eye tissues. Host inflammatory and immune responses to this parasite and antigens from the mf and adult worm stages result in major and sometimes severe pathologies; which include intense itching, dermatitis, atrophy, visual impairment and blindness [3, 4]. Host response to antigens of *Wolbachia* (the endosymbiont of *Onchocerca*) has also been associated with eye pathology [5].

Current efforts to control onchocerciasis are almost exclusively dependent on controlling transmission using mass distribution of ivermectin. This has also been successful in reducing the public health burden of both ocular and dermal manifestations of the disease with >65.3% global coverage [6]. However, sole reliance on ivermectin is not without its limitations as recent reports indicate continuous evolution of ocular onchocerciasis even after 17 years of consistent ivermectin treatment [7]. Furthermore, apart from being only a microfilaricide, unresponsiveness of *O*. *volvulus* to ivermectin treatment in some areas of West Africa have emerged [8]. As a result, attempts to use ivermectin in combination with the antibiotic doxycycline have been made, resulting in long-term sterility of adult female worms and a corresponding absence of mf [9, 10]. Other studies have shown the possibility of achieving macrofilaricidal activity after 6 weeks treatment with 200 mg/day doxycycline [11, 12]. Though significant macrofilaricidal activity has been observed with doxycycline [13–15], a prolonged (6 weeks) daily treatment serves as contraindication in pregnant or breastfeeding women, and children <8 years old [16]. This impedes the use of this class of anti-*Wolbachia* drugs in mass drug administration. Other drugs like albendazole, flubendazole, and oxytetracycline have been implicated in the treatment of onchocerciasis [17, 18] with oxytetracycline and flubendazole showing good macrofilaricidal activity against *O*. *ochengi* [13, 19]. However, wider testing of flubendazole in humans was restricted 30 years ago, as it was poorly tolerated and poorly absorbed from the gut given its formulation [2, 20]. In addition, its intramuscular route of administration results in painful sterile abscesses. Recently, Janssen announced the discontinuation of the development of an orally bioavailable formulation (amorphous solid dispersion) of flubendazole due to safety concerns [21, 22]. Given the aforementioned attributes and serious limitations of the current drugs, it is unlikely that eradication, or even stable elimination, can be achieved without a safe and effective macrofilaricide.

The potential development of such a macrofilaricide is mired by the lack of an appropriate pre-clinical small laboratory animal model to evaluate drug candidates *in vivo* [23]. The only small laboratory animal model developed and validated for adult *O*. *ochengi* reported till date is the SCID (Severe Combined Immuno-Deficient) mouse model, which is immunodeficient [24]. In this model, the role of the acquired immunity in parasite killing is not considered, necessitating more suitable models. In the SCID mice model, viable adult male worms were recovered 35 days post-implantation [24], indicating that the male worms can survive long enough to see the effect of slow acting compounds. For over 2 decades, the Mongolian gerbil *(Meriones unguiculatus)* has been known to have a uniquely high susceptibility to various parasitic infections, including filarial parasites [25]. It is currently used as an animal model for *Brugia* and *Litomosoides sigmodontis* [26–28] and has also been implicated in the study of giardiasis and schistosomiasis [29, 30]. Furthermore, immunocompetent gerbil has recently been shown to harbor adult male *O*. *ochengi* worms for 42 days but drugs which directly target the worms were not used in the study [31]. Given the long-standing use of gerbils as a model for other parasitic infections, we investigated it as a model for testing and validating new macrofilaricidal drugs against *O*. *ochengi* adult male worms.

## Methods

### Ethics statement

The animal protocol (UB-IACUC N^o^ 002/2017) was approved by the University of Buea Animal Care and Use Committee, following recommendations from the ‘Guide for the Care and Use of Laboratory Animals’, 8^th^ edition by the National Research Council, USA. This *in vivo* study was reported in accordance with the ARRIVE Guidelines for reporting animal research.

### Experimental animals

Male and female Mongolian gerbils (*Meriones unguiculatus*, (≥ 6 weeks old)) were purchased from Charles River (France). These animals were maintained in a conventional animal house at the Biotechnology Unit of the University of Buea, and given food and water *ad libidum*. Using the ClinCalc sample size calculator, the minimum number of animals suggested was 4 animals per group. However, the IACUC regulations require that the minimum number of animals able to give meaningful statistics be used and therefore we used at least 5 animals (3 exceptionally for the positive control group) per group in respect of these basic tenets.

### Isolation of *O*. *ochengi* adult male worms from cattle skin and implantation in gerbils

Worms were isolated from umbilical cattle skin by the method of Cho-Ngwa *et al*., (2010) [32]. Fresh pieces of cattle skin with palpable nodules were purchased from a local slaughter house, washed repeatedly with tap water and rinsed with distilled water. The skin was towel-dried, sterilized with 70% ethanol, and allowed to dry in a laminar flow hood. Using a razor blade, the pale orange-yellow *O*. *ochengi* adult worm masses were excised with care not to hurt the adult worm. The entire mass was subsequently submerged in incomplete culture medium (ICM); RPMI-1640 (SIGMA, USA), supplemented with 25 mM HEPES, 2 g/L sodium bicarbonate, 2 mM L-glutamine, 200 units/ml penicillin, 200 μg/ml streptomycin and 0.25 μg/ml amphotericin B, pH 7.4 in 24-well tissue culture plates. Damaged worms and worms from putrefied nodules were discarded. The plates containing the worms were then incubated at 37°C in humidified air with 5% CO_2_ overnight allowing male worms to emerge. Thereafter, the viability of the male worms was evaluated microscopically using an inverted microscope (Nikon Eclipse TS100) before implantation. For implantation, the gerbils were sedated with 80/5 mg/kg body weight ketamine/xylazine. The abdominal area of each animal was shaved and sterilized with 70% ethanol. Using a size 23 sterile surgical blade (Everich, China), a small incision (~1 cm) was made on the skin and peritoneal membrane of each animal.

Twenty (20) male worms were implanted in the peritoneum of each gerbil. Using a sterile nylon monofilament non-absorbable suture needle from Medtrue Enterprise (Nigeria), interrupted stitches were made. The peritoneal membrane was stitched separately from skin, to avoid displacement of the worms from the peritoneum. The stitched area was sterilized with 70% ethanol; animals were placed in cages containing freshly sterilized beddings and monitored daily.

### Drug administration

Flubendazole (FBZ) (cat# 34091) and oxfendazole (OFZ) (cat# 34176) were obtained from Sigma-Aldrich (Germany). Both drugs were administered subcutaneously, with FBZ given at a dose of 10 mg/kg body weight, SID, and OFZ given at 12 mg/kg body weight, BID, for 5 days starting 3 days post-implantation. The vehicle used for both drugs was composed of 0.5% HEC (hydroxyethyl cellulose), 0.1% Tween 80, and 99.4% water.

### Recovery of *O*. *ochengi* adult worms from gerbils

Gerbils were sacrificed via cranial dislocation 35 days post-implantation of *O*. *ochengi* adult male worms. After opening the peritoneum, the cavity was thoroughly observed for the presence of adult male worms, either free or in nodules. Male nodules found in the peritoneum were introduced into collagenase B at a concentration of 0.5 mg/ml and incubated for at least 2 hours at 37°C. After incubation, all worms were observed under the microscope and observations recorded. For all confirmatory experiments, analyses of motility were performed in a blinded manner, where animal groups were only decoded after all the worms had been recovered and scored.

### Assessing the viability of *O*. *ochengi* male worms recovered from gerbils

The motility of adult male worms was evaluated using an inverted microscope. Motility scores ranged from 100% (no observable reduction in motility), 90% (very minute reduction in motility), 75% (slight reduction in motility), 50% (general sluggish movement), and 25% (movement in only head or tail), to 0% (no movement).

Viability of male worms was assessed by MTT [3-(4, 5-dimethylthiazol-2-yl)-2, 5-diphenyl tetrazolium bromide] staining. Each recovered male worm was incubated in 500 μl of 0.5 mg/ml MTT] for 30 min, transferred onto an absorbent paper, and electronic images captured against a white background. The individual male worms were then decolorized by incubating each at 37°C in 200 μl of DMSO for 30 min. The optical density (OD) was measured at 595 nm using a microplate reader (Molecular Devices, USA).

### Data analyses

Data were analyzed using GraphPad Prism 5.0 software and statistical analyses were performed using the Mann-Whitney non-parametric test for two groups of data or Kruskal-Wallis test with Dunn’s multiple comparison post-test for three groups of data. Data presented show mean ± SEM. Data were considered significant if the differences were p <0.05.

## Results

### Ratio of *O*. *ochengi* adult male worms to worm masses

**Fig 1:**
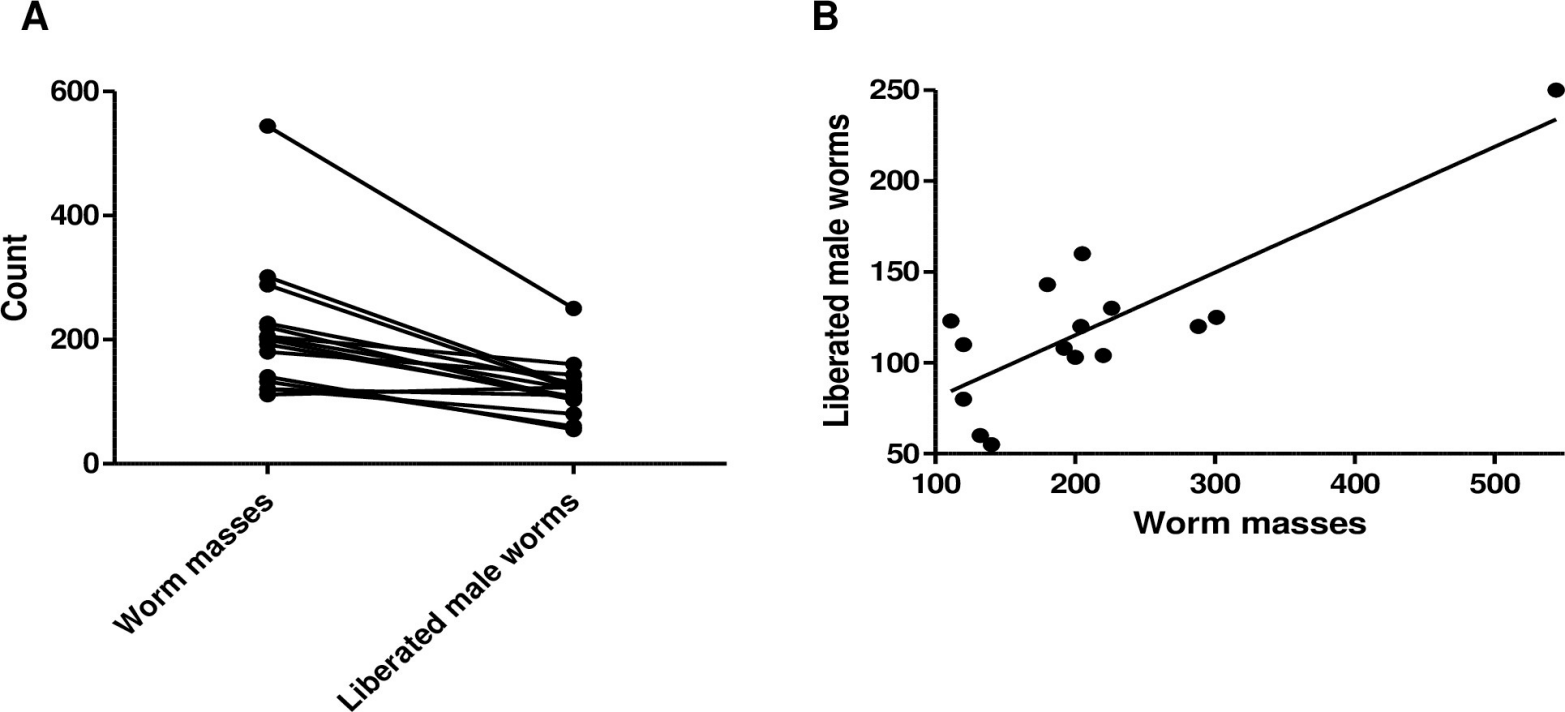
Correlation between the number of worm masses and the number of liberated adult *O*. *ochengi* male worms. Total number of worm masses obtained from cattle skin each time (n = 15) and the corresponding number of liberated male worms, A) Paired comparison of the number of worm masses and number of liberated male worms, B) Correlation between the number of worm masses to liberated male worms. Line shows linear regression best fit (Pearson r = 0.8171; p = 0.0002).

Cattle skin naturally infected with *O*. *ochengi* was obtained from slaughter houses in Douala, Littoral Region and Buea, South West Region of Cameroon. Each visit to the slaughter house yielded an average of 212.2 ± 28.0 (range: 111–544) worm masses from infected cattle skin, from which an average of 127.6 ± 12.7 (range: 55–250) adult male worms were recovered. Except for one case, the number of male worms obtained was always less than the number of worm masses recovered (Fig 1A). The average ratio of worm masses to liberated adult male worms was 1:0.6. The number of liberated male worms correlated with number of worm masses from which they emerged, Pearson r = 0.8171; p = 0.0002 (Fig 1B), although a single data point was much higher than the rest of the data. Based on our experience, on average, ~350 worm masses can be harvested per week which yields ~210 adult male worms.

### Comparable recovery of *O*. *ochengi* adult male worms in male and female gerbils

**Fig 2:**
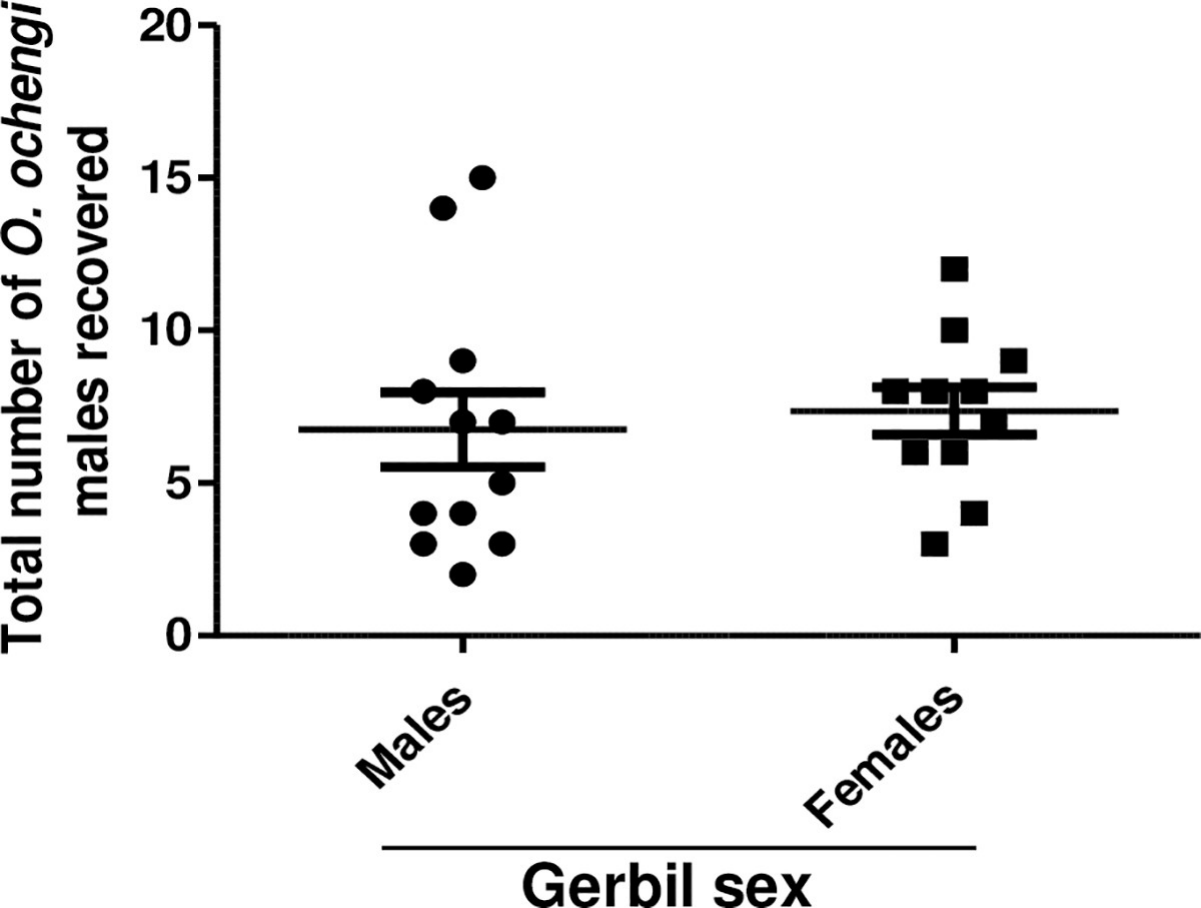
Comparison of *O*. *ochengi* male worm recovery in male and female gerbils. Gerbils were implanted with 20 *O*. *ochengi* males, sacrificed 35 days later and analyzed for total number of male worms recovered in male and female gerbils (n = 11–12 per group).

To see if there was a sex bias in the survival of adult *O*. *ochengi* male worms in gerbils, both male and female animals were infected with 20 male worms. Gerbils were analyzed 35 days post-implantation. It was found that 6.8 ± 1.2 adult male worms were recovered from male gerbils, while 7.3 ± 0.8 worms were recovered from female gerbils (Fig 2). Thus, the survival of *O*. *ochengi* adult male worms in male and female gerbils was not significantly different, allowing for both sexes to be used indiscriminately in this study. Considering both male and female gerbils, the average recovery was 7.0 ± 0.8 which is equivalent to 35%.

### *O*. *ochengi* adult male worms in gerbil peritoneum were either moving freely or encapsulated in nodules

**Fig 3:**
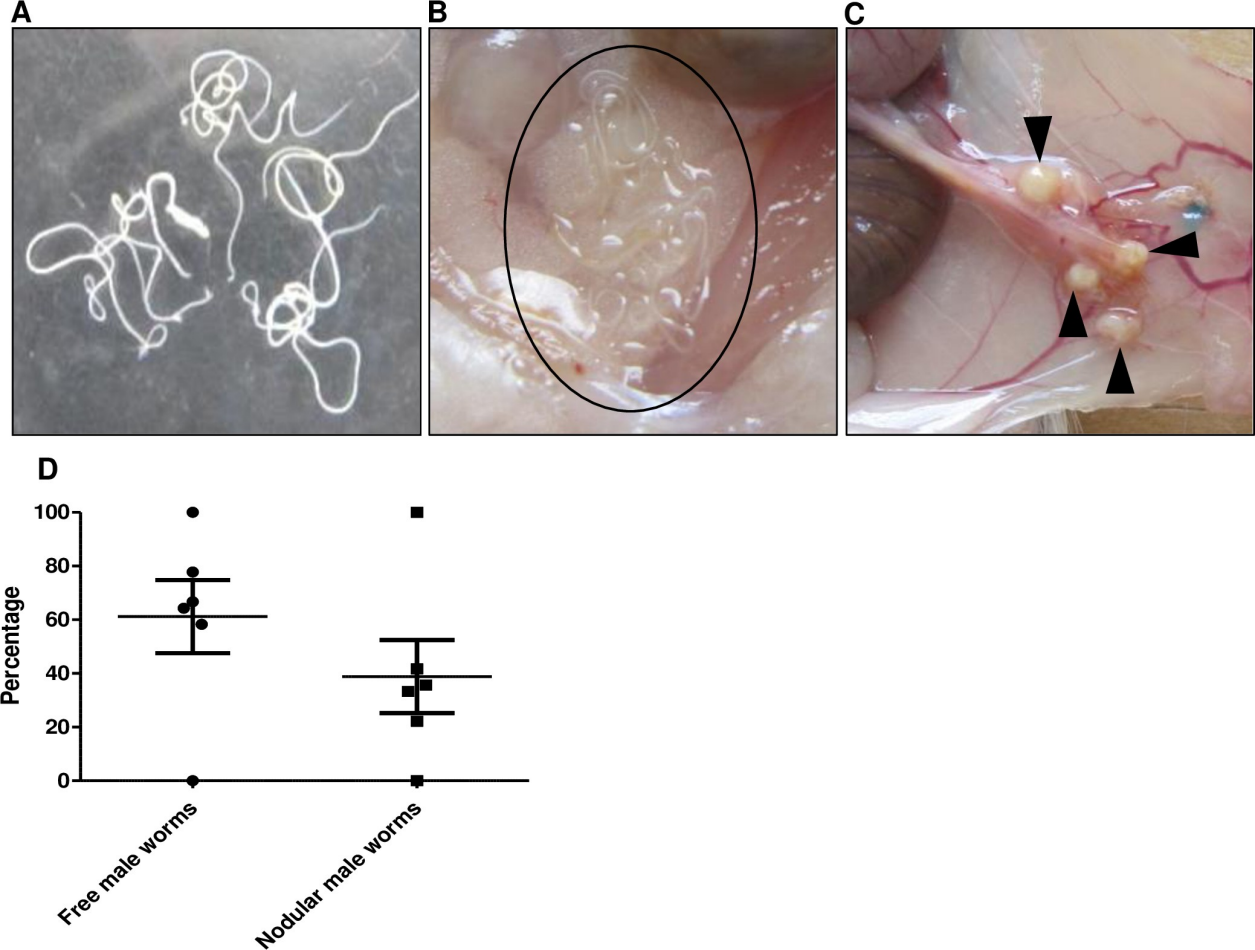
Recovery of free or nodular *O*. *ochengi* adult male worms within the peritoneum of gerbils. A) Liberated *O*. *ochengi* adult male worms *in vitro* from worm masses removed from infected cattle skin, B) Cluster of free *O*. *ochengi* adult male worms in the peritoneum of gerbils (circle) 35 days post-implantation, C) Nodules containing *O*. *ochengi* adult male worms in the peritoneum of gerbils (arrow head) 35 days post-implantation, D) Percentage of free and nodular *O*. *ochengi* adult male worms in the peritoneum of gerbils 35 days post-implantation. Analyses showed that 61.2 ± 13.6% of the male worms recovered were free in the peritoneum while 38.8 ± 13.6% were in nodules (Fig 3D). There was no statistically significant difference in the motility of male worms recovered from nodules or found free in the peritoneum of control groups (S1 Table). Eighty nine (89)% of the *O*. *ochengi* male worms recovered from the exploratory gerbils scored 100% motility.

Fig 3A shows *O*. *ochengi* adult male worms that emerged *in vitro* from *O*. *ochengi* worm masses after 24 hours in culture. Upon sacrifice of untreated gerbils 35 days post-implantation, it was noticed that some of male worms were found free and could be seen moving in the peritoneum (Fig 3B) while other male worms were encapsulated within newly formed nodules (Fig 3C). The encapsulated male worms were released by digestion of the nodules with collagenase. Both free and nodular *O*. *ochengi* male worms had no preferred location and could be found anywhere in the peritoneum.

### Validation of the gerbil *O*. *ochengi* adult male worm model with flubendazole (FBZ)

**Fig 4:**
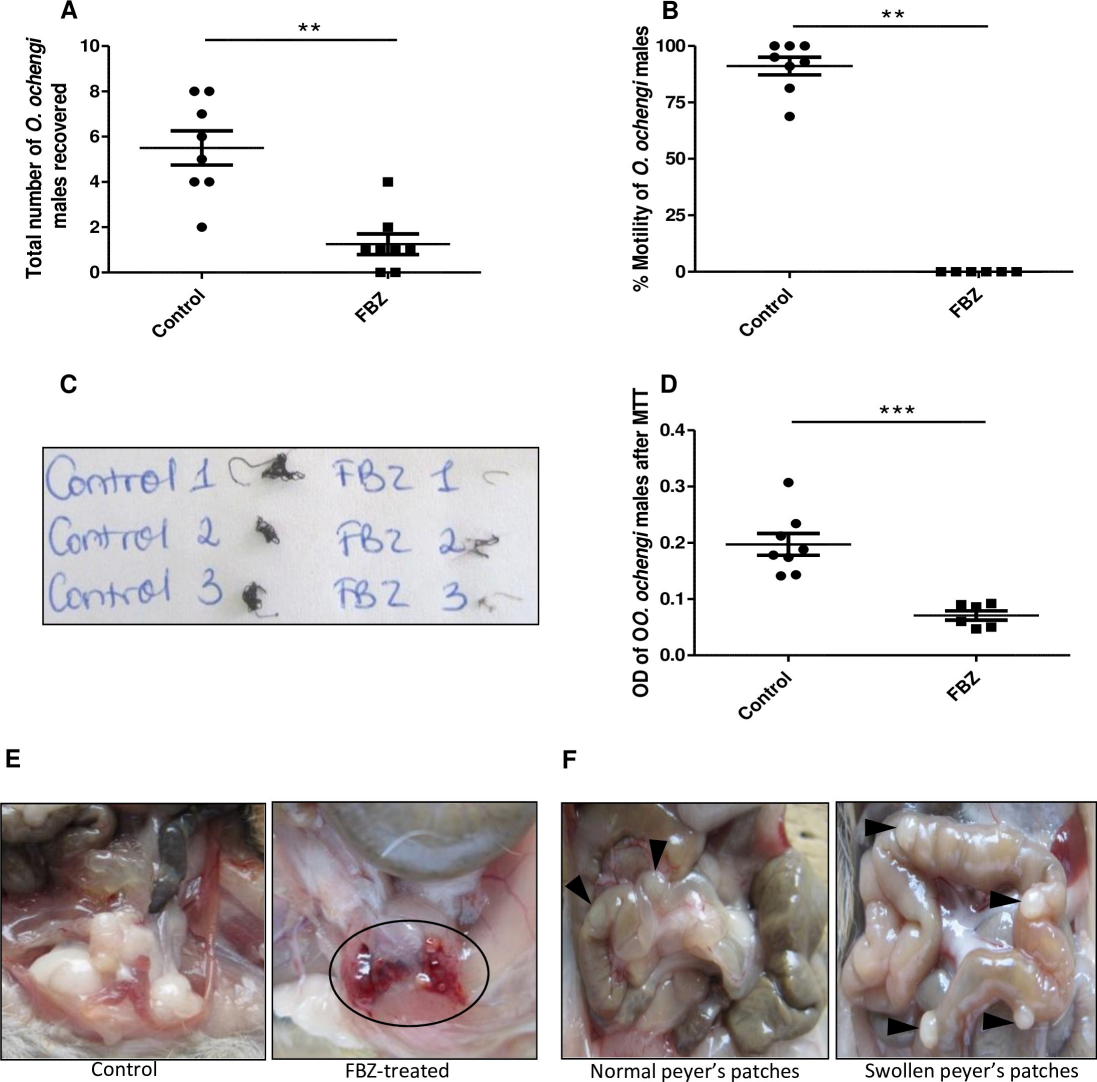
Flubendazole (FBZ) decreases *O*. *ochengi* male worm burden and motility in gerbils. Gerbils were implanted with 20 *O*. *ochengi* male worms and treated for 5 days subcutaneously starting on day 3 post-implantation with 10 mg/kg FBZ, SID. Animals were sacrificed on day 35 post-implantation. A) Total number of male worms recovered, B) Percentage motility of the male worms recovered, C) Photo of *O*. *ochengi* male worms from control and FBZ-treated gerbils after MTT staining, D) OD of DMSO treated male worms after MTT staining, E) Blood clot in the lower peritoneal cavity of FBZ-treated animal (circle), and F) Picture of normal and swollen Peyer’s patches (arrow head). Results are representative of five independent experiments. N = 8 per group. Statistical significance was done by Mann Whitney test. **p <0.01; ***p <0.001.

To validate the model, we treated gerbils implanted with *O*. *ochengi* adult male worms with the potent macrofilaricide; FBZ, and compared with vehicle-treated gerbils. FBZ treatment significantly decreased (p = 0.0021) the number of recovered male worms in gerbils (1.3 ± 0.5), compared to the number (5.5 ± 0.8) in control animals (Fig 4A). In addition, there was a complete inhibition of the motility of the recovered adult male worms from the FBZ-treated gerbils whereas the motility in control animals was 91.1 ± 3.9%, p = 0.0019 (Fig 4B). Pictures of MTT-stained adult male worms from control animals clearly showed that the worms were viable as they stained dark blue as opposed to worms from FBZ-treated animals which had a faint blue coloration (Fig 4C). Measuring the OD of DMSO used to decolorize MTT-stained male worms, showed a significantly reduced OD (p = 0.0007) for worms from FBZ-treated animals (0.07 ± 0.01) compared to worms from control gerbils (0.20 ± 0.02) (Fig 4D).

More so, 80% (16/20) of FBZ-treated gerbils had blood clots in their abdominal cavities (Fig 4E) while such clots were not observed in control gerbils (0/18). Some gerbils had very prominent Peyer’s patches (Fig 4F). Though not significantly different (p = 0.4392), the occurrence of prominent Peyer’s patches was higher in the FBZ-treated group; 67% (6/9), compared with the control group; 43% (3/7).

### Oxfendazole (OFZ) significantly reduces worm motility

**Fig 5:**
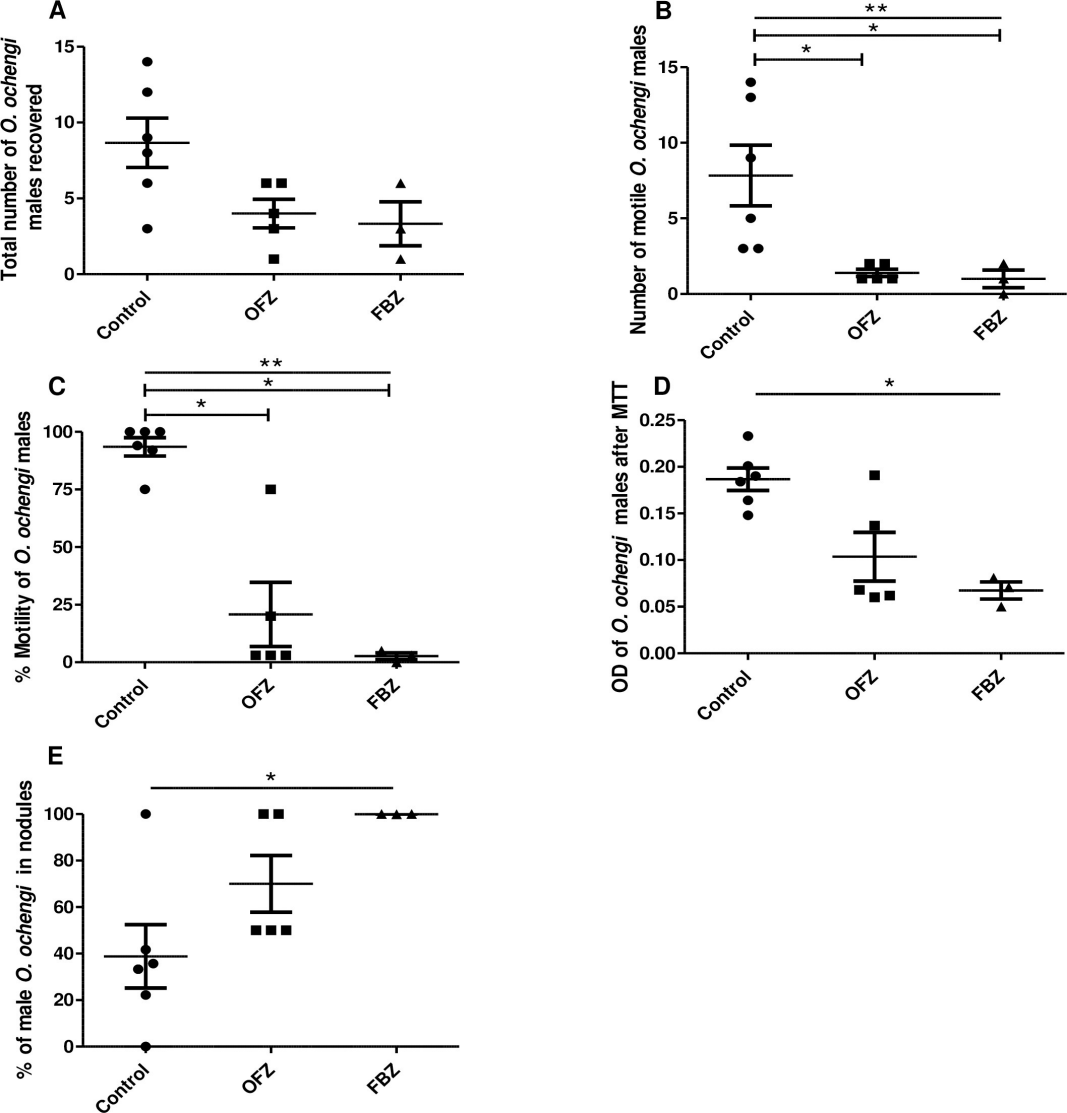
Oxfendazole (OFZ) decreases the number of *O*. *ochengi* motile male worms and their viability. Gerbils were implanted with 20 *O*. *ochengi* male worms and treated for 5 days starting on day 3 post-implantation subcutaneously with 12 mg/kg OFZ, BID, or with 10 mg/kg FBZ, SID. Animals were sacrificed on day 35 post-implantation. A) Total number of male worms recovered, B) Number of viable male worms recovered, C) Percent motility of the male worms recovered, D) OD of DMSO treated male worms after MTT staining, and E) Percent of *O*. *ochengi* male worms within the nodules. FBZ was used as positive control. N = 3–6 per group. Statistical significance between groups was done using Kruskal-Wallis Test with Dunn’s multiple comparison. *p <0.05; **p <0.01.

Using the validated *O*. *ochengi* adult male worm gerbil model, we tested the effect of OFZ; a drug similar to FBZ, that is used as an anthelmintic in livestock. A total of 8.7 ± 1.6 male worms were retrieved from the control gerbils while 4.0 ± 1.0 were found in OFZ-treated animals (Fig 5A). Of the total male worms recovered, 7.8 ± 2.0 were motile in the control group, which was significantly higher (p = 0.0073), compared to the 1.4 ± 0.2 motile worms recovered from the OFZ-treated group (Fig 5B). The percent motility of worms (93.0 ± 4.0) in the control group was equally significantly higher (p = 0.0097), compared to the worms in the OFZ-treated group (20.0 ± 14.0) (Fig 5C). Similarly, the OD after MTT staining of *O*. *ochengi* male worms from control gerbils (0.19 ± 0.01) was higher (but not significant; p = 0.0519), compared to worms from OFZ-treated gerbils (0.10 ± 0.03) (Fig 5D). The percent male worms within nodules in the OFZ-treated group was 70.0 ± 12.2 as opposed to 38.8 ± 13.6 (p = 0.0530) in the control group (Fig 5E). FBZ was used as positive control and its effects on worm burden and viability were more severe, compared with OFZ-treated gerbils (Fig 5A–5D). Interestingly, all the worms recovered in the FBZ-treated group were retrieved from newly formed nodules (Fig 5E). The OFZ-treated gerbils equally had blood clots in the peritoneum, similar to FBZ-treated animals.

## Discussion

In this study a small animal model for the screening of macrofilaricide activity against onchocerciasis was developed. Mongolian gerbils were implanted with *O*. *ochengi* adult male worms and shown to be a validated model for testing known macrofilaricidal drugs such as flubendazole. Such a model represents a great contribution in onchocerciasis preclinical drug development, as drug candidates identified using *in vitro* screening assays can now be tested in this model. This will also allow a better prediction of the activity of new drugs against the human *O*. *volvulus in vivo*. The macrofilaricide; FBZ, was used to validate this model. The burden of adult male *O*. *ochengi* worms implanted in FBZ-treated gerbils was assessed on day 35 post-implantation and showed a significant decrease (78%); the number of recovered worms was 5.5 ± 0.8 in control gerbils and 1.3 ± 0.5 in FBZ-treated gerbils (Fig 4A). Remarkably, worm motility was completely inhibited in the FBZ-treated gerbils, but 91.1 ± 3.9% in the controls (Fig 4B). Using this model, OFZ used in livestock as an anthelmintic drug, was equally tested. Although there was no significant difference in the total number of worms recovered from the animals in each group (Fig 5A), in the OFZ-treated gerbils, the recovered viable worms had significantly reduced motility *(*p = 0.0073) (Fig 5C). This gerbil model is the first adult *Onchocerca* model described using immunocompetent animals and therefore promises to be a very robust model. Further analyses are required to better understand the role of the immune system in this model, which is probably also drug-dependent.

The effect of drugs on the parasite depends on many variables including the parasite species, the specific host, the drug concentration, formulation, route of administration, and other assay conditions. In addition, PK/PD varies from one host to another. Moreover, the difference in the location of the parasite within its natural human host and in this particular gerbil model (peritoneum) may also lead to some differences in pharmacokinetics and therefore, drug efficacy. Based on our results, OFZ is not as good as FBZ although it was given at a higher dose (twice daily at 12 mg/kg body weight while FBZ was given once daily at 10 mg/kg). Interestingly, in OFZ- and FBZ-treated gerbils, almost all the recovered worms were non-motile (Figs 4B and 5C).

Although a SCID mouse model of *O*. *ochengi* adult male worms has equally been described [24], our study used gerbils that are immunocompetent, and thus the possible synergistic involvement of the acquired immune response to the killing of the parasites can equally be evaluated in this model [31]. Ivermectin and diethylcarbamazine (DEC) have been shown to have no effect or require much higher concentrations for microfilaricidal activity *in vitro* than *in vivo* [33–35] supporting a synergistic role between the drug activity and the immune system. Interestingly, 43% of the control gerbils developed swollen Peyer’s patches after male *O*. *ochengi* implantation, indicating some involvement of the immune response; related to the effect of the drugs on the male worms, and/or because of the release of parasite proteins once the worms are killed. Gerbils have also been shown to have not only cellular, but also humoral immune responses to filarial antigens [36, 37].

The susceptibility of the gerbils to implanted male *O*. *ochengi* was not sex dependent as comparable numbers of worms were retrieved from both males and females (Fig 2). This makes it easier to get sufficient number of animals for experiments as both sexes can be used indiscriminately. However, survival of certain filarial nematodes has been shown to be animal sex dependent. For example, male gerbils are more susceptible to *B*. *malayi* than females [38]. Another nematode, *L*. *sigmondontis*, survives better in female than in male Balb/c mice [39].

This gerbil model with *O*. *ochengi* male worms we have developed is a good model for screening especially slow acting (requiring 30 or more days to act) drugs. The results obtained on day 35 post-implantation showed 89% of the male worms at 100% viability. This high viability indicates that the worms can survive beyond this time point. Killing the male worms will prevent the fertilization of female eggs, thereby preventing mf production. Although killing the adult female worm is preferred, such a model for *O*. *ochengi* is not yet available. Results from our laboratory showed that female *O*. *ochengi* worms do not survive for long in rodents commonly used in laboratory studies, including gerbils. The only published report of viable female *O*. *ochengi* worms was in SCID mice sacrificed 7 days post-implantation [24]. Even though female *O*. *volvulus* survives in SCID mice or athymic nude rats for up to 20 weeks post-implantation [40], such a model cannot be used for routine screening. Although closely related, *O*. *ochengi* and *O*. *volvulus* may present with different sensitivities. However, the closeness in phylogeny has proven to work in the past with ivermectin for example, clearing *O*. *ochengi* microfilariae in cattle, with the same efficiency with which it clears *O*. *volvulus* microfilariae in humans. *O*. *ochengi* has also been shown to share over 95% conserved regions with *O*. *volvulus* [41], this presents with a very slim chance of a potential drug molecule not to have similar effects in both species. More so, it will be too costly to get enough *O*. *volvulus* worms from humans for routine screens.

Interestingly, 38.8 ± 13.6% of the free male worms implanted in untreated animals were found in newly formed nodules, while 61.2 ± 13.6% of the male worms recovered were free in the peritoneum (Fig 3D). Although it is still unclear why some of these worms induce the formation of nodules in the peritoneum, the immune system would most likely play a role in worm recovery, and the formation of the nodule could be the result of an immune response to the wall of the infection, and/or response to the angiogenic factors the implanted worms secrete. The viable worms recovered from the nodules were generally as motile as the free worms found in the peritoneum (S1 Table). We did not find any reason that could suggest that formation of the nodules was linked to degradation of dead worms. It is therefore important to do a meticulous peritoneal search when analyzing the animals, to avoid missing any free males. Furthermore, the free males may be more exposed and thereby more susceptible to the drugs, as 100 ± 0.0% and 70.0 ± 12.2% of worms retrieved from FBZ- and OFZ-treated animals respectively, were recovered from nodules, as opposed to 38.8 ± 13.6% recovered from nodules in control animals (Fig 5E).

FBZ and OFZ belong to the class of benzimidazoles which are aromatic organic compounds used for decades as anthelmintics [42]. These β-tubulin binding drugs inhibit worm motility, reproduction and cell secretory processes [43, 44]. FBZ was very effective in validating this model (Fig 4A–4D). This is in line with observations by Halliday *et al*., (2014). In addition, a modified FBZ (UMF-078) was shown to be effective against infection with *O*. *ochengi* in naturally infected cattle [45]. Using the present gerbil model, it was found that OFZ had significant activity on *O*. *ochengi* male worms (Fig 5), howbeit less efficient than FBZ. OFZ was administered twice daily at 12 mg/kg body weight while FBZ was given once daily at 10 mg/kg, but both had similar activity, if not higher with FBZ. In recent studies [46] comparing the *in vitro* activity of FBZ and OFZ on *O*. *volvulus*, L5s have equally shown OFZ to be less effective. For instance, both drugs presented a similar IC_50_ (0.13 μM) for the inhibition of motility, which differed when the inhibition of viability was determined; 4 times higher with OFZ, compared with FBZ (0.54 μM vs. 0.13 μM). This further proves that the kinetics or activity is different for both drugs and may equally demand optimized *in vivo* timing. Perhaps for drugs like OFZ, a longer time point (>35 days) will result in complete killing of the male worms. With the recent good tolerance shown by OFZ in a Phase 1 study [47], it remains a promising drug to treat onchocerciasis in humans.

In conclusion, we have developed and validated an immunocompetent gerbil *O*. *ochengi* male worm model to aid the development of novel *Onchocerca* macrofilaricides. Using this model, we found that OFZ, a drug belonging to the same class as FBZ, was equally a potential macrofilaricidal drug.

## Supporting information

S1 Table Viability of the free vs nodular worms recovered from gerbils. All free worms were scored for motility before the nodular ones. The general motility score was similar in all the worms (almost always 100%). Gerbil SN 4 and 5 clearly illustrate this with 100% motility of all worms recovered, whether free or from nodules (p = 0.0931).
(PPTX)
