# Supplementary material for: Development and validation of an Onchocerca ochengi adult male worm gerbil model for macrofilaricidal drug screening
Source: PLoS Negl Trop Dis. 2019 Jul 1;13(7):e0007556. doi: 10.1371/journal.pntd.0007556 (PMC6625737; doi:10.1371/journal.pntd.0007556)
Supplement: S1 Table — All free worms were scored for motility before the nodular ones. The general motility score was similar in all the worms (almost always 100%). Gerbil SN 4 and 5 clearly illustrate this with 100% motility of all worms recovered, whether free or from nodules (p = 0.0931). (PPTX) [file pntd.0007556.s001.pptx]

## Slide 1
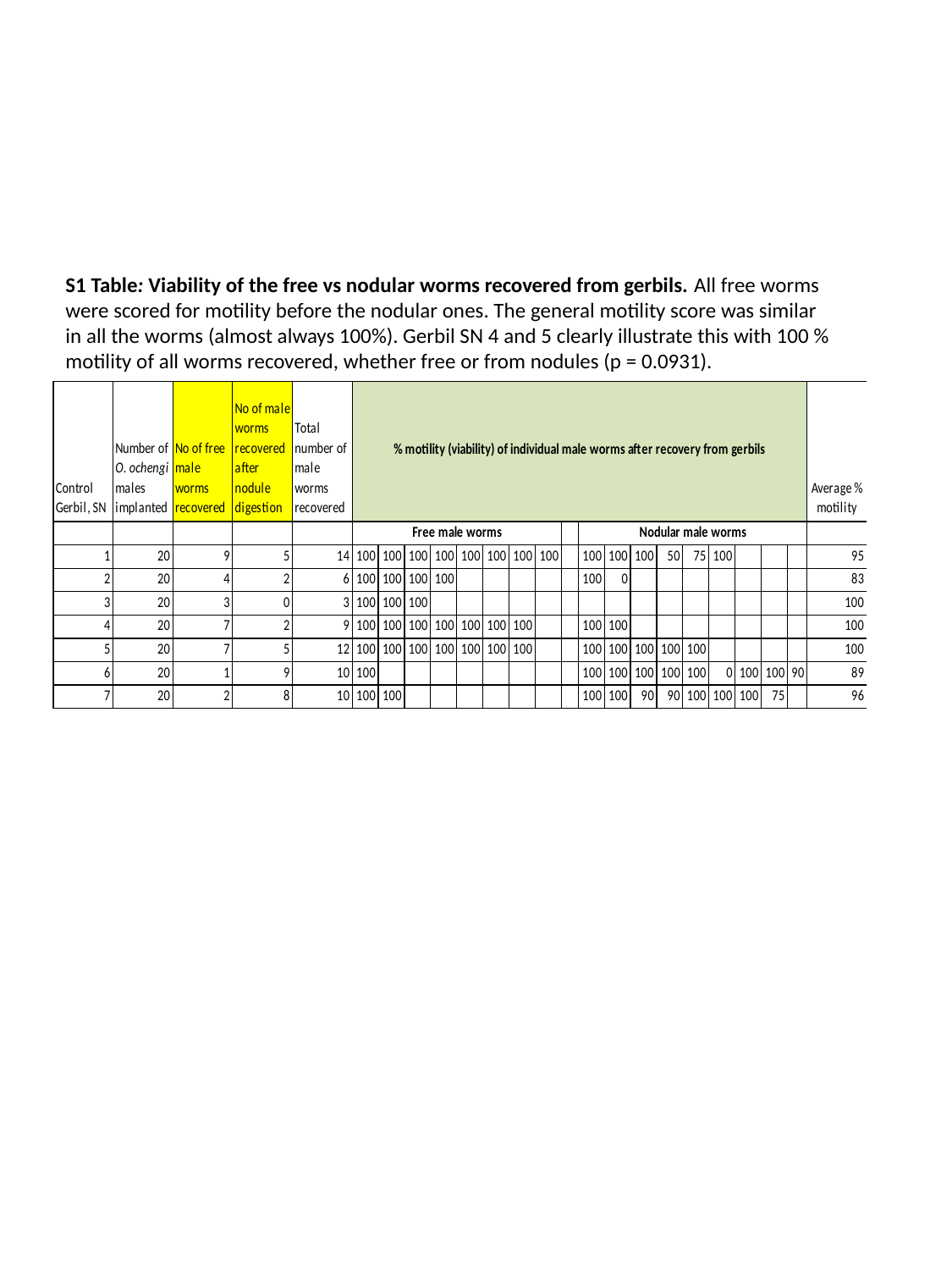

S1 Table: Viability of the free vs nodular worms recovered from gerbils. All free worms were scored for motility before the nodular ones. The general motility score was similar in all the worms (almost always 100%). Gerbil SN 4 and 5 clearly illustrate this with 100 % motility of all worms recovered, whether free or from nodules (p = 0.0931).
